# Hepatitis B Virus Reactivation During Rituximab Therapy Despite Anti-HBs Positivity: A Case Report

**DOI:** 10.7759/cureus.110047

**Published:** 2026-06-01

**Authors:** Rajae Belhocine, Nawal Lagdali, Maryeme Kadiri, Borahma Mohamed, Fatima Zohra Ajana

**Affiliations:** 1 Department of Hepato-Gastroenterology C, Ibn Sina Hospital, Mohammed V University in Rabat, Rabat, MAR; 2 Department of Gastroenterology C, Ibn Sina Hospital, Mohammed V University in Rabat, Rabat, MAR

**Keywords:** anti-hbs, hbv reactivation, hepatitis b virus, immunosuppression, lymphoma, rituximab

## Abstract

Hepatitis B virus (HBV) reactivation is a well-recognized complication of immunosuppressive therapy, particularly with B-cell-depleting agents such as rituximab. Anti-HBs positivity may reflect previous immune control, but it does not eliminate the risk of reactivation in patients with previously resolved HBV infection. We report the case of a 63-year-old man with splenic marginal zone lymphoma treated with rituximab-based chemotherapy. Before treatment, HBV testing showed negative HBsAg, positive anti-HBc, positive anti-HBs, and undetectable HBV DNA. No antiviral prophylaxis was initiated before chemotherapy, although the patient was retrospectively considered at high risk for HBV reactivation because of anti-HBc positivity and planned rituximab therapy. Approximately two months after the last chemotherapy cycle, he developed marked hepatic cytolysis. Further evaluation showed new HBsAg positivity, persistent anti-HBs positivity, and detectable HBV DNA, confirming HBV reactivation with HBsAg seroreversion. Tenofovir disoproxil fumarate was started, resulting in normalization of liver enzymes and sustained virological suppression. This case emphasizes that anti-HBs positivity should not be considered sufficient protection against HBV reactivation in patients receiving rituximab. Systematic HBV screening, guideline-based antiviral prophylaxis in high-risk patients, and prolonged virological monitoring are essential to prevent severe hepatic complications.

## Introduction

Hepatitis B virus (HBV) reactivation is a potentially severe complication of immunosuppressive therapy [1-3]. It may occur in both patients with chronic HBV infection and those with resolved HBV infection because HBV can persist within hepatocytes as covalently closed circular DNA. When immune control is impaired, viral replication may resume, leading to hepatitis, liver failure, interruption of chemotherapy, or death [1-3].

For general readers, HBV serological markers are important for understanding this risk. HBsAg indicates current HBV infection, anti-HBc reflects previous or ongoing exposure to HBV, and anti-HBs usually indicates humoral immunity after vaccination or resolved infection. However, anti-HBs positivity does not fully exclude the risk of HBV reactivation during profound immunosuppression, particularly in patients receiving B-cell-depleting therapy. Rituximab, an anti-CD20 monoclonal antibody, is one of the agents most strongly associated with HBV reactivation [4-7]. By inducing prolonged B-cell depletion, it can impair humoral immune control and increase the risk of reactivation, even in HBsAg-negative and anti-HBc-positive patients [4-7]. Therefore, patients with resolved HBV infection who receive rituximab-based therapy require careful risk assessment, antiviral prophylaxis when indicated, and prolonged virological monitoring.

We report a case of HBV reactivation in a patient with splenic marginal zone lymphoma treated with rituximab-based chemotherapy despite baseline anti-HBs positivity. This case highlights that anti-HBs positivity alone should not be considered sufficient protection in high-risk patients and emphasizes the importance of systematic HBV screening, guideline-based antiviral prophylaxis, and prolonged follow-up.

## Case presentation

A 63-year-old man with type 2 diabetes mellitus and arterial hypertension was diagnosed with stage IV splenic marginal zone lymphoma with hepatic and bone marrow involvement, confirmed by liver biopsy. The initial presentation was marked by left upper quadrant heaviness, with no documented B symptoms. He was a former smoker with a 20 pack-year history and had stopped smoking 18 years earlier. He had no history of blood transfusion, no HBV vaccination, and no known chronic liver disease.

As part of the pretreatment evaluation, liver tests were within normal ranges, with AST 24 IU/L, ALT 33 IU/L, alkaline phosphatase 60 IU/L, gamma-glutamyl transferase 38 IU/L, and total bilirubin 12 µmol/L. Prothrombin time was also normal at 93%. HBV serology showed negative hepatitis B surface antigen (HBsAg), positive anti-HBc, and positive anti-HBs, with an anti-HBs titer of 66.3 IU/L, a profile consistent with previously resolved HBV infection. Baseline HBV DNA was undetectable. Hepatitis C virus and HIV serologies were negative. No antiviral prophylaxis was initiated before chemotherapy, although, in retrospect, the patient met high-risk criteria for HBV reactivation because of anti-HBc positivity and planned rituximab-based therapy.

The patient received six cycles of rituximab, cyclophosphamide, vincristine, and prednisone (R-CVP) between January and May 2023. The documented cycle dates were January 3, January 27, February 16, March 17, April 17, and May 17, 2023. Interim computed tomography showed a 65% decrease in lymphadenopathy size and regression of splenomegaly, consistent with a partial response. The end-of-treatment assessment performed on June 22, 2023, showed no residual abnormality, and the biological evaluation was unremarkable, consistent with complete clinical and paraclinical remission.

Approximately two months after the last chemotherapy cycle, severe hepatic cytolysis was incidentally detected during follow-up. Laboratory tests showed aspartate aminotransferase (AST) 554 IU/L, approximately 16 times the upper limit of normal; ALT 1149 IU/L, approximately 20 times the upper limit of normal; gamma-glutamyl transferase 196 IU/L; alkaline phosphatase 68 IU/L; and total bilirubin 12 µmol/L, while prothrombin time remained preserved at 80%. The patient had no jaundice and no clinical signs of liver failure. The longitudinal biochemical and virological profile is summarized in Table 1.

**Table 1:** Longitudinal biochemical and virological profile. AST: Aspartate aminotransferase; ALT: Alanine aminotransferase; HBsAg: Hepatitis B surface antigen; anti-HBs: Hepatitis B surface antibody; HBV: Hepatitis B virus.

Time point	AST/ALT	Total bilirubin	HBsAg	anti-HBs	HBV DNA
Pretreatment evaluation before rituximab	AST 24 IU/L; ALT 33 IU/L	12 µmol/L	Negative	Positive, 66.3 IU/L	Undetectable
HBV reactivation, late July/early August 2023	AST 554 IU/L; ALT 1149 IU/L	12 µmol/L	Positive	Positive, 65.81 IU/L	796,596.99 IU/mL; 5.90 log IU/mL
Follow-up, January 2024	Normalized	Normalized	Not documented	Not documented	Undetectable
Last follow-up, July 29, 2024	AST 18 IU/L; ALT 13 IU/L	4.7 µmol/L	Not documented	Not documented	Undetectable

Repeat HBV testing performed in late July 2023 showed new HBsAg positivity after previously negative baseline HBsAg, consistent with HBsAg seroreversion in the setting of HBV reactivation. Anti-HBc remained positive, and anti-HBs was still detectable, with an anti-HBs titer of 65.81 IU/L. HBeAg and anti-HBe were negative on available testing. HBV DNA quantification performed on August 1, 2023, showed a viral load of 796,596.99 IU/mL, corresponding to 5.90 log IU/mL. Anti-HCV and HIV serologies remained negative. Based on new HBsAg positivity, detectable HBV DNA, and marked hepatic cytolysis after rituximab-based chemotherapy, a diagnosis of rituximab-associated HBV reactivation was established.

Tenofovir disoproxil fumarate 300 mg/day was started on August 2, 2023, with close clinical, biochemical, virological, and renal monitoring. At follow-up in January 2024, the patient was clinically asymptomatic, liver tests had normalized, and HBV DNA was undetectable. At the last available follow-up on July 29, 2024, nearly 12 months after tenofovir initiation, the patient remained asymptomatic, with sustained normalization of liver tests: AST 18 IU/L, alanine aminotransferase (ALT) 13 IU/L, gamma-glutamyl transferase 64 IU/L, alkaline phosphatase 56 IU/L, and total bilirubin 4.7 µmol/L. HBV DNA remained undetectable. Renal monitoring showed an estimated glomerular filtration rate of 65 mL/min and serum phosphorus of 34 mg/L. The evolution of HBsAg serology was not documented, and follow-up was continued every three months.

During follow-up, isolated anti-M2 antibody positivity was detected. However, a diagnosis of primary biliary cholangitis was not retained because the patient fulfilled only one diagnostic criterion, namely anti-M2 antibody positivity. There was no persistent cholestasis, and liver histology did not show characteristic biliary lesions. Sequential liver biopsies showed moderate inflammatory activity (A2) and portal fibrosis (F2) according to the METAVIR scoring system, without ductal lesions or specific inflammatory findings.

## Discussion

HBV reactivation is a well-recognized complication of immunosuppressive therapy and may occur in both patients with chronic HBV infection and individuals with previously resolved infection. It results from the loss of immune control over residual intrahepatic HBV, leading to renewed viral replication. In HBsAg-negative and anti-HBc-positive patients, HBV reactivation may be defined by detectable HBV DNA, with or without HBsAg seroreversion and transaminase elevation [1-3]. The present case fulfills these criteria, with new HBsAg positivity consistent with HBsAg seroreversion, detectable HBV DNA, and marked hepatic cytolysis after rituximab-based chemotherapy.

Rituximab is one of the immunosuppressive agents most strongly associated with HBV reactivation. By binding to the CD20 antigen, it induces prolonged B-cell depletion and may impair humoral immune memory, including the persistence and functional efficacy of anti-HBs antibodies [4-6]. Consequently, even HBsAg-negative and anti-HBc-positive patients may experience viral replication from residual intrahepatic HBV genetic material after exposure to rituximab. This mechanism explains why patients with resolved HBV infection remain at risk under B-cell-depleting therapy, regardless of anti-HBs positivity [1-7].

Recent international recommendations, including the 2025 European Association for the Study of the Liver (EASL) Clinical Practice Guidelines, as well as American Association for the Study of Liver Diseases (AASLD), Asian Pacific Association for the Study of the Liver (APASL), and American Society of Clinical Oncology (ASCO) guidance, emphasize systematic HBV screening before immunosuppressive or anticancer therapy, using at least HBsAg and anti-HBc, with anti-HBs and baseline HBV DNA helping to further characterize the risk profile. In HBsAg-negative and anti-HBc-positive patients receiving anti-CD20 monoclonal antibodies such as rituximab, antiviral prophylaxis is recommended because these patients are considered at high risk for HBV reactivation, even when baseline HBV DNA is undetectable. Although an anti-HBs level >10 IU/L is traditionally considered a marker of serological protection, this threshold may be insufficient during profound immunosuppression. Higher anti-HBs titers, including ≥100 IU/L, have been suggested as possible markers of lower reactivation risk, but no anti-HBs threshold should replace guideline-based antiviral prophylaxis in HBsAg-negative and anti-HBc-positive patients receiving rituximab. This concept is illustrated by our case, in which HBV reactivation occurred despite baseline anti-HBs positivity and undetectable baseline HBV DNA [1-3,7].

The present case illustrates this concept. Before chemotherapy, our patient had negative HBsAg, positive anti-HBc, and positive anti-HBs, with an anti-HBs titer of 66.3 IU/L. This level was above the conventional threshold of 10 IU/L but below the >100 IU/L threshold proposed as more relevant in high-risk immunosuppressed patients. Despite this apparently reassuring serological profile, the patient developed HBV reactivation after rituximab-based chemotherapy. This observation confirms that anti-HBs positivity alone should not be considered sufficient protection against HBV reactivation in patients exposed to B-cell-depleting therapies.

Prevention of HBV reactivation relies on four major steps: comprehensive HBV screening, risk stratification according to the type of immunosuppression, appropriate antiviral prophylaxis, and prolonged monitoring [1-3]. Universal screening before chemotherapy or immunotherapy should include HBsAg, anti-HBc, and anti-HBs testing. This strategy allows identification of patients with chronic or resolved HBV infection and helps determine the appropriate preventive approach before the initiation of immunosuppressive treatment.

Risk stratification is mainly based on the immunosuppressive regimen. High-risk situations include anti-CD20 antibodies, such as rituximab, obinutuzumab, and other B-cell-depleting therapies, as well as hematopoietic stem cell transplantation. Intermediate-risk therapies include anti-TNF agents, prolonged high-dose corticosteroids, and some conventional chemotherapy regimens, whereas lower-risk situations include methotrexate, azathioprine, or short courses of low-dose corticosteroids [1-3].

In patients at high risk of HBV reactivation, antiviral prophylaxis with a nucleos(t)ide analogue with a high genetic barrier to resistance is recommended. Entecavir, tenofovir disoproxil fumarate, and tenofovir alafenamide are the preferred agents [1-3]. Antiviral therapy should ideally be started before immunosuppression, usually one to two weeks before the first cycle, and continued after completion of therapy. In the case of rituximab and other anti-CD20 antibodies, prolonged prophylaxis is required because B-cell depletion may persist for months after treatment cessation [1-3].

Post-treatment monitoring remains essential, particularly in patients exposed to high-risk therapies such as rituximab. Follow-up should include regular assessment of liver enzymes, HBsAg status, and HBV DNA, generally every three to six months, and should be prolonged for at least 12 months after the end of immunosuppressive therapy [1-3,7]. In the present case, follow-up included clinical assessment, liver tests, HBV DNA monitoring, and renal tolerance monitoring under tenofovir. The patient was asymptomatic in January 2024, with normalization of liver tests and undetectable HBV DNA, and remained asymptomatic at the last available follow-up on July 29, 2024, nearly 12 months after tenofovir initiation, with sustained biochemical normalization and undetectable HBV DNA. However, HBsAg evolution during follow-up was not documented, which represents a limitation of the longitudinal serological monitoring.

The effectiveness of antiviral prophylaxis has been demonstrated in several studies and reviews, showing a significant reduction in HBV reactivation, severe hepatitis, and HBV-related mortality among patients receiving immunosuppressive therapy [7-10]. Conversely, a strategy based solely on monitoring may expose patients to unpredictable and potentially severe reactivation, particularly in the context of rituximab therapy.

In addition to hepatology and oncology recommendations, the WHO 2024 guidance emphasizes the identification and protection of high-risk groups, including immunocompromised patients. It supports systematic screening, expanded access to antiviral therapy, and prevention of HBV-related complications, in line with the principles proposed by international liver societies [11].

Overall, this case reinforces the importance of systematic antiviral prophylaxis in anti-HBc-positive patients receiving anti-CD20 therapy, regardless of anti-HBs positivity. In clinical practice, prevention of HBV reactivation should be considered a standard of care in patients exposed to significant B-cell-depleting immunosuppression. An integrated approach combining pretreatment screening, appropriate antiviral prophylaxis, and prolonged virological monitoring remains essential to prevent severe and potentially life-threatening post-rituximab HBV reactivation.

## Conclusions

HBV reactivation remains a potentially severe complication in patients receiving rituximab-based therapy, even in those with resolved HBV infection and anti-HBs positivity. This case highlights that anti-HBs positivity does not fully eliminate the risk of HBV reactivation during B-cell-depleting therapy. Therefore, HBsAg-negative and anti-HBc-positive patients receiving rituximab should be considered at high risk and should receive guideline-based antiviral prophylaxis, regardless of anti-HBs status. Systematic HBV screening, appropriate prophylaxis, and prolonged virological monitoring remain essential to prevent clinically significant HBV reactivation.
